# Association of diurnal temperature range with daily hospitalization for exacerbation of chronic respiratory diseases in 21 cities, China

**DOI:** 10.1186/s12931-020-01517-7

**Published:** 2020-09-29

**Authors:** Zihui Wang, Yumin Zhou, Ming Luo, Huajing Yang, Shan Xiao, Xiaoliang Huang, Yubo Ou, Yongbo Zhang, Xianzhong Duan, Wei Hu, Chenghao Liao, Yijia Zheng, Long Wang, Min Xie, Longhui Tang, Jinzhen Zheng, Sha Liu, Fan Wu, Zhishan Deng, Heshen Tian, Jieqi Peng, Xinwang Wang, Nanshan Zhong, Pixin Ran

**Affiliations:** 1 State Key Laboratory of Respiratory Disease, National Clinical Research Center for Respiratory Disease, Guangzhou Institute of Respiratory Health, the First Affiliated Hospital of Guangzhou Medical University, Guangzhou Medical University, Guangzhou, China; 2 School of Geography and Planning, Sun Yat Sen University, Guangzhou, China; 3 Government Affairs Service Center of Health Commission of Guangdong Province, Guangzhou, China; 4 Guangdong Environmental Monitoring Center, Guangzhou, China; 5 Guangdong Provincial Academy of Environmental Science, Guangzhou, China; 6 Department of Ecology and Environment of Guangdong Province, Guangzhou, China

**Keywords:** Diurnal temperature range, Chronic obstructive pulmonary disease, Asthma, Bronchiectasis, Hospitalization

## Abstract

**Background:**

The association between diurnal temperature range (DTR) and hospitalization for exacerbation of chronic respiratory diseases (CRD) was rarely reported.

**Objectives:**

To examine the association between DTR and daily hospital admissions for exacerbation of CRD and find out the potential effect of modifications on this association.

**Method:**

Data on daily hospitalization for exacerbation of chronic obstructive pulmonary disease (COPD), asthma and bronchiectasis and meteorology measures from 2013 through 2017 were obtained from 21 cities in South China. After controlling the effects of daily mean temperature, relative humidity (RH), particulate matter < 2.5 μm diameter (PM_2.5_) and other confounding factors, a standard generalized additive model (GAM) with a quasi-Poisson distribution was performed to evaluate the relationships between DTR and daily hospital admissions of CRD in a two-stage strategy. Subgroup analysis was performed to find potential modifications, including seasonality and population characteristics.

**Result:**

Elevated risk of hospitalization for exacerbation of CRD (RR = 1.09 [95%CI: 1.08 to 1.11]) was associated with the increase in DTR (the 75th percentile versus the 25th percentile of DTR at lag0–6). The effects of DTR on hospital admissions for CRD were strong at low DTR in the hot season and high DTR in the cold season. The RR (the 75th percentile versus the 25th percentile of DTR at lag0–6) of hospitalization was 1.11 (95%CI: 1.08 to 1.12) for exacerbations of COPD and 1.09 (95%CI: 1.05 to 1.13) for asthma. The adverse effect of DTR on hospitalization for bronchiectasis was only observed in female patients (RR = 1.06 [95%CI: 1.03 to 1.10]).

**Conclusion:**

Our study provided additional evidence for the association between DTR and daily hospitalization for exacerbation of CRD, and these associations are especially stronger in COPD patients and in the cold season than the hot season. Preventive measures to reduce the adverse impacts of DTR were needed for CRD patients.

## Introduction

High prevalence of chronic respiratory diseases (CRD) has contributed to the magnitude of the non-fatal health burden globally [1]. Chronic obstructive pulmonary disease (COPD) is the fourth leading cause of mortality in the world, especially in the elderly population, and the third leading cause of years of life lost in China [2]. Asthma is also one of the most common CRD in high-income areas with a global prevalence of 4.3% (95% CI: 4.2 to 4.4) in adults [3]. Given great prevalence in both developed and developing countries, bronchiectasis is also regarded as one of the most common chronic respiratory diseases [4]. Acute exacerbations of CRD refer to episodes of worsening symptoms and commonly resulted in seeking healthcare use, including outpatient service, emergency room visits, and hospital admissions [5]. As one of the severe outcomes of exacerbations, hospitalization is a major contributor to the disease burden of CRD [6].

Climate change, usually caused by human activity, was suggested to be risk factors of health effects, especially in CRD [7]. Diurnal temperature range (DTR), as defined by the difference between the maximum and minimum temperatures within 1 day, is an important meteorological indicator associated with climate change [8]. Previous studies showed a positive association between DTR and non-CRD hospitalization [9–11]. Moreover, gender, age, season and geographical location may modify the effects of DTR on mortality which indicated that some subpopulations are more susceptible to DTR than others [10, 12–15]. For example, mortality among the elderly, the less educated, females were associated more strongly with DTR [16, 17]. Lee et al. suggested the DTR-effect on respiratory mortality was observed in extremely cold region [18]. But the argument about the modifiers of the DTR and CRD-related hospitalization still exists. Lim et al. suggested that the asthma admission was significantly higher in the elderly than those aged under 75 years in short-term DTR exposure, but no significant difference was found in patients with COPD [10]. But Phosri et al. suggested that no significant difference was found when stratified by sex or age in extremely high DTR [9]. Further research is needed to confirm whether those factors (i.e., sex, age, season) will modify the association between DTR and hospitalization.

Moreover, previous studies have been conducted in a focus on a single city and then have omitted the spatial effects of DTR [8]. Applicability of those studies may be limited on multi-city or country scale [19]. Moreover, those studies estimated only a single disease of respiratory rather than CRD [20, 21].

To fill the gaps listed above, we estimated the association of DTR with hospital admissions for exacerbations of CRD in 21 cities, China, from 2013 to 2017. We also evaluated whether the associations were modified by sex, age and seasons (i.e., ‘hot’ and ‘cold’ season).

## Methods

### Meteorological and air pollution data

Guangdong Province is located in the South of China. Our study was limited to 21 cities of Guangdong Province (179,700 km^2^) - Zhanjiang, Maoming, Yangjiang, Zhaoqing, Shaoguan, Heyuan, Meizhou, Qingyuan, Yunfu, Shantou, Shanwei, Chaozhou, Jieyang, Shenzhen, Zhuhai, Foshan, Jiangmen, Dongguan, Zhongshan, Huizhou and Guangzhou. Data on daily maximum, minimum and mean temperatures and relative humidity (RH) were collected from the National Meteorological Information Center of China (http://data.cma.cn/). There were 68 local weather stations recorded daily measures across Guangdong Province from January 1, 2013 to December 31, 2017. We calculated city-wide meteorological measures by averaging data from stations located in a specific city. Without local weather stations, data of Chaozhou city and Foshan city were collected from the nearest monitoring sites located in Jiexi districts and Gaoyao districts, respectively. DTR was calculated by subtracting daily minimum temperature from daily maximum temperature. Data on daily city-wide concentrations of particulate matter < 2.5 μm diameter (PM_2.5_) were obtained from the Guangdong Provincial Environmental Monitoring Center. All data from 102 central monitoring stations in 21 cities were available.

### Hospitalization data

In China, only second and tertiary level hospitals that are qualified to provide specialized medical-care for exacerbation of COPD, asthma and bronchiectasis.

There were 227 government-tiered second or tertiary hospitals in 21 cities that have uploaded their daily hospitalization records to the electronic medical record system of Guangdong Government Affairs Service Center. International Classification of Diseases 10th (ICD-10) codes including J44, J45–46 and J47 were used to identified hospitalization for exacerbations of COPD, asthma and bronchiectasis, respectively.

### Statistical analysis

Spearman correlation analysis was performed among DTR, daily mean temperature, RH and daily concentration of PM_2.5_. The association of DTR with daily hospitalization for exacerbation of CRD was estimated by a two-stage analysis using 21-city data.

In the first stage, we adopted a standard generalized additive model (GAM) with a quasi-Poisson distribution [22] to investigate the city-specific relationship of DTR and hospital admissions for exacerbations of CRD. Seven-day moving average (lag 0–6) was used to present the lag effect of DTR. In the framework of distributed lag non-linear model (DLNM) function, we used a cubic spline for DTR and its lag with 5 and 4 degrees of freedom, respectively. The 25th percentile of DTR is regarded as the centering point. We used a natural cubic function with 8 degrees of freedom per year to control the long-term trends of years and seasonality [23, 24]. Day of the week and the official holiday were included as an indicator to remove the effect of short-term fluctuation [25]. Confounding meteorological measures, including daily mean temperature at lag 0–14, RH at lag 0–3 [26, 27] and daily concentration of PM_2.5_ at lag 0–3 was also included into the GAM as follows:

Log [E (Yt)] = α + βDTR_0–6t_ + ns (time, df = 8/per year) + day of the week + holiday + ns (temperature_0–14t_, df = 3) + ns(RH_0-3t_, df = 3) + ns(PM_2.5 0-3t_, df = 3).

Where E(Yt) presents the daily hospital admissions for exacerbation of CRD on day t; α is the intercept in specific-region; β is the regression coefficient, and its exponent value indicates the relative risk of hospitalization per unit increase of DTR; DTR_0–6t_ is 7-day moving average of DTR; time presents the long term trend (from 1 to 1826); temperature_0–14t_ is 15-day moving average of daily mean temperature; RH_0-3t_ is 4-day moving average of daily relative humidity; PM_2.5 0-3t_ indicates 4-day moving average of daily concentrations of PM_2.5_; ns() presents natural cubic function.

In the second stage, we pooled the estimates in 21 cities by performing a meta-analysis. Considering the heterogeneity of city-level estimate, we adapted the random-effects model by maximum likelihood (REML) rather than fixed effects to ensure a more robust estimation.

To explore the seasonal pattern of relationship between DTR and hospitalization for exacerbation of CRD, data was stratified by time: hot season (May to October) and cold season (November to April of the next year). We used a natural cubic function with 4 degrees of freedom within each 6-month subperiod to control the variation of long-term trends [28]. We also divided the hospitalization data into diverse CRD groups to investigate the heterogeneity of associations of DTR with COPD, asthma and bronchiectasis, respectively. Subgroup analyses on sex and age (i.e., < 65 vs. ≥65 years old) were performed to identify vulnerable population.

### Sensitivity analysis

Several sensitivity analyses were applied to evaluate the robustness of main results: (1) altering numbers of moving average for DTR; (2) varying df of the long-term trend and meteorological measures; (3) replacing the natural cubic spline function by penalized splines function; (4) excluding data of cities with ≤5 hospitals in records.

Analyses were performed in STATA (version 12, StataCorp, TX) and R (version 3.6.2, R Development Core Team) with “mgcv” and “mvmeta” packages. Statistically significance was determined as a two-side *P* value < 0.05.

## Results

### Data description

From January 1, 2013 to December 31, 2017, a total of 670,832 hospital admissions for CRD, including exacerbations of COPD, asthma and bronchiectasis, were obtained in our studies. E-Fig. 1 presents 21 cities in Guangdong Province. During our study period, the minimum of DTR in 21 cities was 2.0 °C and the maximum of DTR was 15.7 °C. The IQR increase of DTR was 4.0 °C (range: 5.0 to 9.0). The mean temperature was 21.9 °C (range: 2.0 to 33.9) in Guangdong Province. The RH was 75.5% (range: 19.1 to 100). The mean of daily hospitalization was 263 (range: 74 to 585) for COPD, 42 (range: 8 to 85) for asthma and 62 (range: 11 to 128) for bronchiectasis (Table 1). The DTR level for each city was presented in e-Table 1. High correlation (i.e., Spearman correlation coefficients>0.7 and *P* value<0.05) was not observed between meteorological and PM_2.5_ (Table 2).

**Fig. 1:**
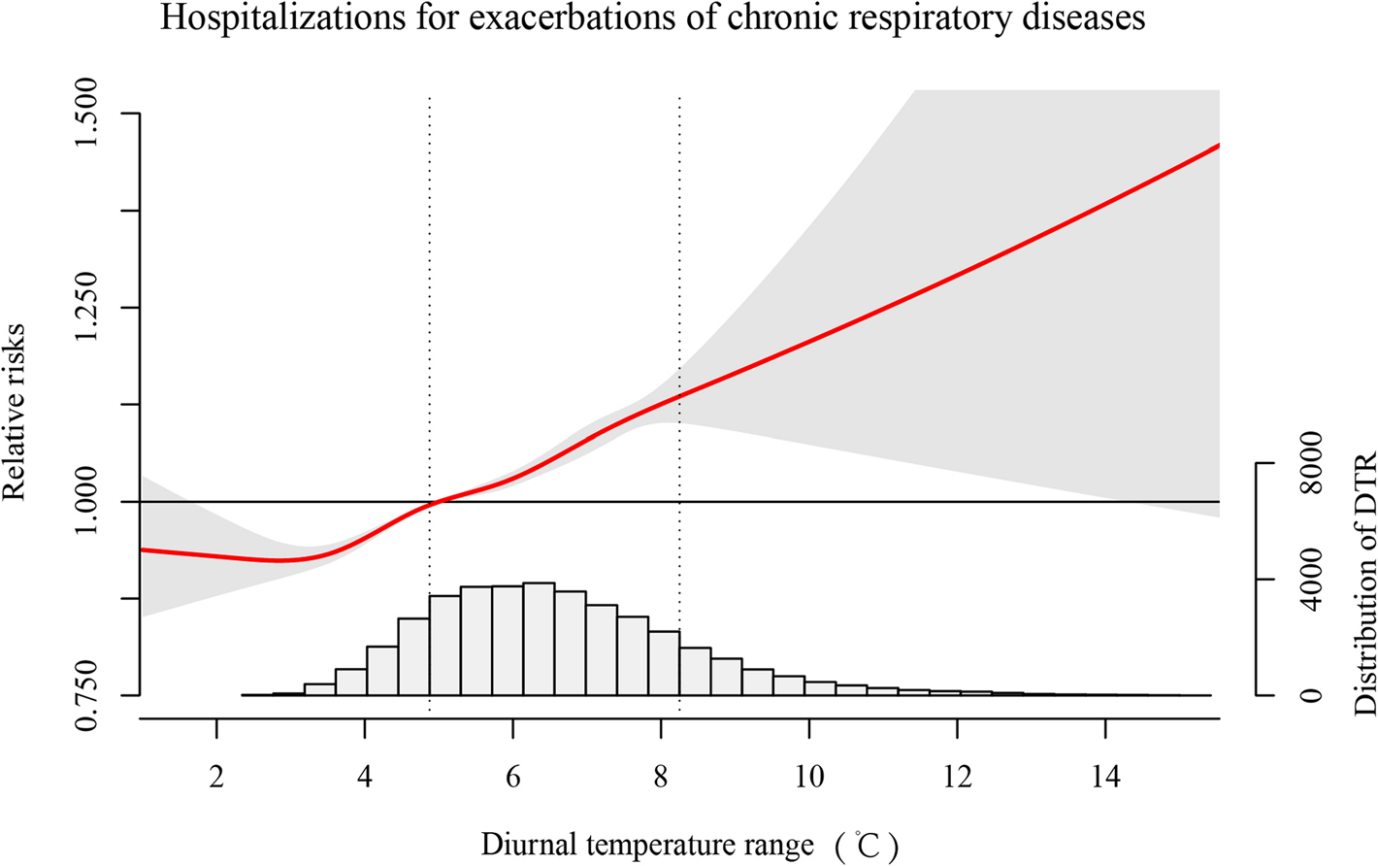
Pooled exposure-response relationship between DTR and hospitalization for exacerbations of chronic respiratory diseases in 21 cities, China during 2013–2017. The pooled curves present by the continuous bold red lines and the grey areas represent the 95% confidence intervals. The vertical dashed lines indicate the interquartile range of DTR (i.e., 25th percentile and 75th percentile) from 2013 through 2017

**Table 1:** City-wide daily meteorological measures, PM_2.5_ and hospital admissions for exacerbations of chronic respiratory diseases in Guangdong Province, 2013–2017

	Mean (SD)	Minimum	Percentile			Maximum
			25th	50th	75th	
Daily meteorology						
Diurnal Temperature Range (°C)	7.1 (3.0)	1.1	5.0	7.0	9.0	15.7
Mean Temperature (°C)	21.9 (6.1)	2.0	17.3	22.8	27.2	33.9
Relative Humidity (%)	75.5 (12.2)	19.1	67.7	76.9	84.5	100.0
PM_2.5_ (μg/m^3^)	37.2 (20.3)	7.1	19.4	32.8	46.1	141.7
Daily hospitalizations						
Chronic Obstructive Pulmonary Disease	263 (76)	74	210	251	309	585
Asthma	42 (11)	8	34	41	49	85
Bronchiectasis	62 (17)	11	50	62	73	128

**Table 2:** Spearman correlation coefficients between meteorological measures and PM_2.5_ in Guangdong Province, 2013–2017

	PM_2.5_	Temperature	Relative humidity	DTR
PM_2.5_	1.00	−0.47*	−0.51*	0.38*
Temperature		1.00	0.19*	0.12*
Relative humidity			1.00	−0.61*
DTR				1.00

* *P* value < 0.005

### Regression results

Figure 1 presents the dose-response relationships between DTR at lag 0–6 days and hospitalization for exacerbation of CRD. The effect of DTR on hospitalization for exacerbation of CRD followed J-shape curves, suggesting that the RRs changed slightly at low level of DTR (range: 1.1 to 4 °C) and increased rapidly with both moderate and high DTR. The RR of hospitalization for exacerbation of CRD was 1.09 (95%CI:1.08 to 1.11) at the 75th percentile compared to the 25th percentile of DTR at lag0–6.

**Fig. 2:**
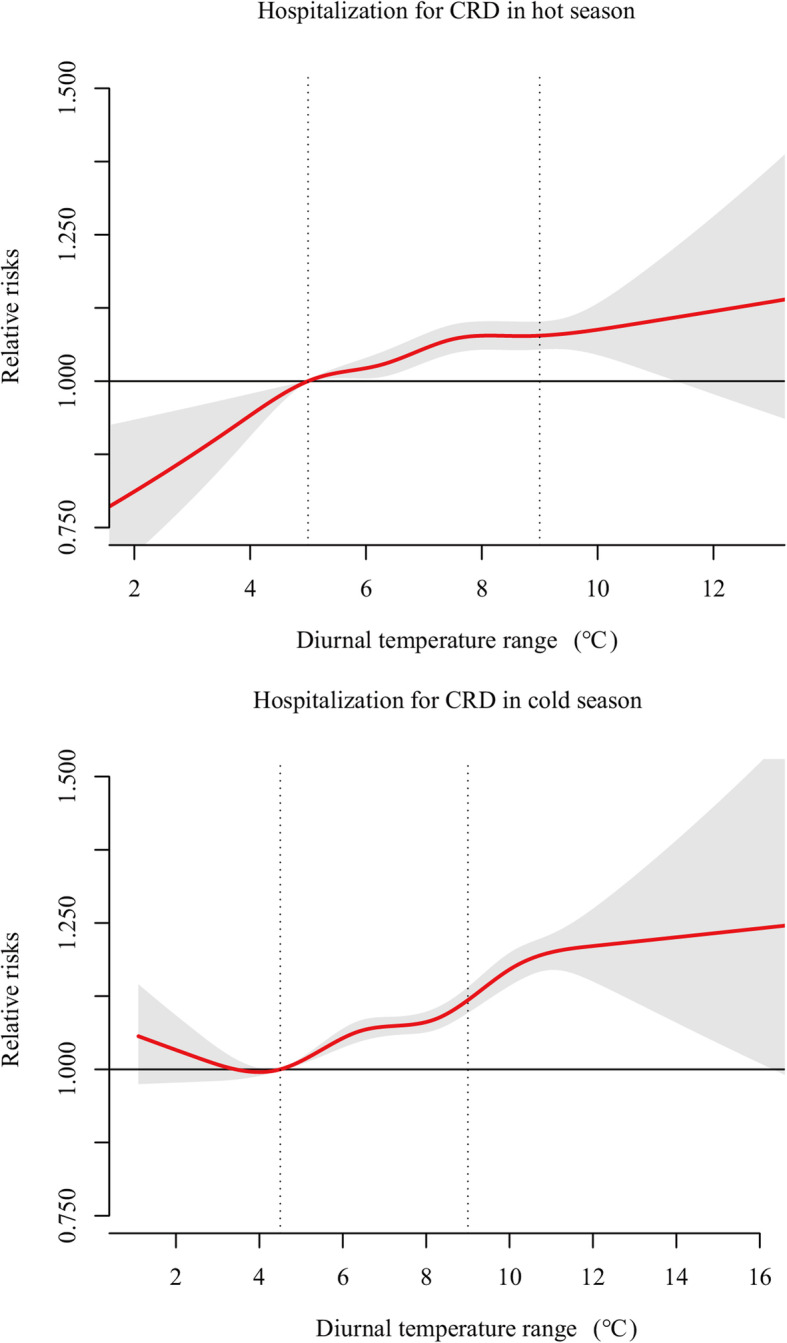
Pooled DTR –hospitalization for Chronic respiratory disease (CRD) association in hot season and cold season. The pooled curves present by the continuous bold red lines and the grey areas represent the 95% confidence intervals. The vertical dashed lines indicate the interquartile range of DTR (i.e., 25th percentile and 75th percentile) in hot season and cold season

Figure 2 shows the season-specific effects of DTR on hospitalization for CRD. In the hot season (May to October), the effect of DTR was rapidly increased from the minimum DTR to 8 °C but slightly increased at DTR above 8 °C. The RR (the 75th percentile vs. the 25th percentile of DTR at lag0–6) of hospitalization was 1.08 (95%CI: 1.05 to 1.11). In the cold season (November to April of the next year), no significant effect was found at DTR below 4.4 °C (i.e., 25 percentile of DTR in cold season), and the RR of hospital admissions climbed sharply at the 75th percentile compared to the 25th percentile of DTR at lag0–6 (RR = 1.11 [95%CI: 1.07 to 1.13]) and increased slowly at DTR > 11 °C.

**Fig. 3:**
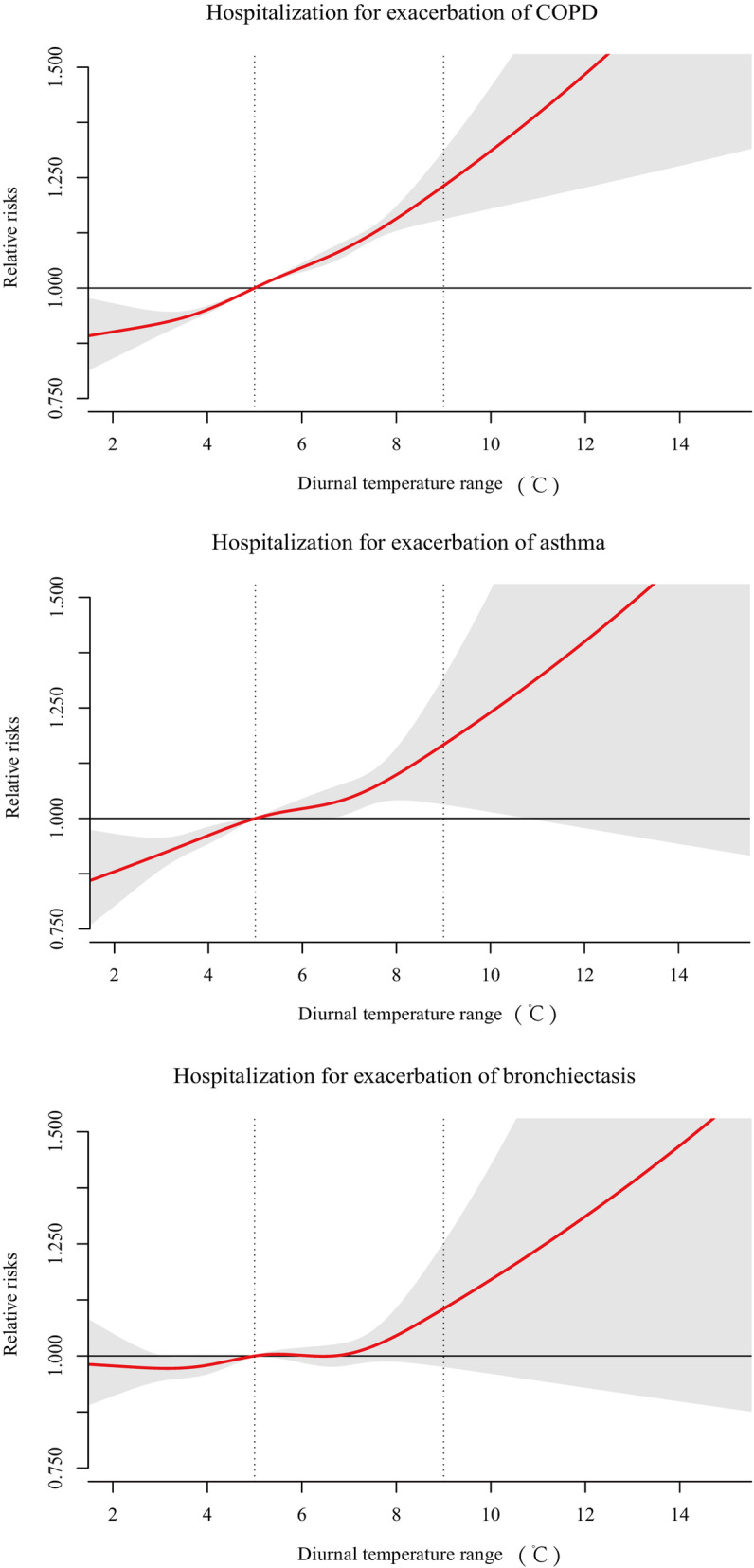
Pooled exposure-response relationship between DTR and hospitalization for exacerbations of chronic obstructive respiratory diseases (COPD), asthma and bronchiectasis in 21 cities, China during 2013–2017. The pooled curves present by the continuous bold red lines and the grey areas represent the 95% confidence intervals. The vertical dashed lines indicate the interquartile range of DTR (i.e., 25th percentile and 75th percentile) from 2013 through 2017

For COPD, the dose-response curve increased constantly in the entire DTR range, with an RR of 1.11 (95%CI: 1.08 to 1.12) (Fig. 3). A positive relationship was also found between DTR and hospital admissions for exacerbations of asthma and the RR was 1.09 (95%CI: 1.05 to 1.13) at the 75th percentile compared to the 25th percentile of DTR at lag0–6. However, statistical significance disappeared at the high level of DTR. For bronchiectasis, the lower 95% CI of the estimate was less than 1 in the whole range of DTR, showing on significant association (Fig. 3).

Table 3 shows the RR of hospitalization for exacerbations of CRD stratified by age (≥65 years and <65 years) and sex (male and female). Associations of hospitalization for COPD and asthma with DTR were found when the analysis stratified by age and sex. In bronchiectasis patients, non-significant association was observed in the aged subgroup or male group, while a positive relationship was found in female patients (RR = 1.06 [95%CI: 1.03 to 1.10]).

**Table 3:** Subgroup analysis based on age, sex and season^a^

	COPD	Asthma	Bronchiectasis
Age			
≥ 65	1.10 (1.05 to 1.14)	1.08 (1.02 to 1.15)	1.05 (0.97 to 1.13)
< 65	1.11 (1.10 to 1.13)	1.08 (1.03 to 1.13)	1.02 (0.89 to 1.13)
Sex			
Male	1.11 (1.08 to 1.14)	1.10 (1.05 to 1.16)	1.01 (0.97 to 1.05)
Female	1.11 (1.08 to 1.13)	1.06 (1.02 to 1.11)	1.06 (1.03 to 1.10)
Season			
Hot season	1.02 (1.00 to 1.04)	1.05 (0.99 to 1.11)	1.00 (0.96 to 1.04)
Cold season	1.09 (1.07 to 1.12)	1.05 (1.01 to 1.09)	1.01 (0.98 to 1.04)

^a^ Results are presented by relative risk (95% CI) at 75th percentile compared to 25th percentile of DTR at lag0–6

City-specific estimates and results related to meta-analysis, such as I^2^ and Q test for heterogeneity from random-effect meta-analysis were presented in e-Table 2.

### Sensitivity analysis

The RR was consistent when we altered numbers of moving average for DTR at lag0–6, lag0–13 and lag0–20. Estimates were also stable when degrees of freedom for long-term trend and meteorological measure changed. We also observed similar results when replaced the natural cubic spline function with the penalized splines function. Estimated RRs remained statistically significant at lag0–6 even when data for Chaozhou, Jieyang, Shantou and Shanwei were excluded (e-Table 3).

## Discussion

After adjusting for confounding factors (i.e., daily mean temperature, RH and PM_2.5_), we confirmed that DTR was an independent risk factor on hospital admissions for exacerbations of CRD, represented by COPD, asthma and bronchiectasis. We also assessed the potential effect modification of season and population characteristics (i.e., sex and age). We found that the associations, as well as the dose-response curves, were diverse in the hot and cold seasons. Hospital admissions for COPD and asthma were associated with DTR. Adverse effects of DTR on bronchiectasis patients were only observed in female.

Most of the previous studies focused on the relationship between DTR and mortality, and respiratory and cardiovascular diseases were regarded as the main cause of mortality after short-term exposure of DTR [29]. Morbidity is another important outcome of exposure to DTR. The effects of DTR on respiratory-related emergency room visit and out-patients service were usually investigated in the previous study but few hospital admissions which reflects severe effects of DTR [30–33]. To fill this knowledge gap, we carried out a province-wide study to investigate the association of hospitalization for exacerbation of CRD, represented by COPD, asthma and bronchiectasis. Our study discovered a nonlinear DTR-CRD relationship in subtropical regions. High RRs of moderate and extreme high (i.e., 50th percentile and 100th percentile) DTR deserved more attention. There are potential mechanisms linking DTR and hospitalization for exacerbation of CRD: 1) the host defense function of the respiratory system, nasal responses and airway mucociliary clearance could be influenced when the temperature of respiratory epithelium fluctuated [34, 35]; 2) increased DTR might enhance the transmission of virus and bacteria and resulted in the occurrence of exacerbations of respiratory diseases [29, 36].

In our study, the maximum lag effect of DTR was identified at lag0–6 (RR = 1.09 [95%CI 1.08 to 1.1]) (e-Table 2). Furthermore, the lag effect persisted even when the number of moving average days increased to 21 days (RR = 1. 09 [95%CI: 1.06 to 1.12]). A previous study also demonstrated that 8 days moving average of DTR was associated with respiratory emergency room admissions [30]. Similar characteristics of the lag effect of DTR measures deserve great attention, especially in severe outcome variables like hospital admission. Identifying the significant effect period for the occurrence of the disease is helpful for the prediction of the DTR-related adverse events [29].

Our study has focused on the whole range of DTR, including extreme DTR in both hot and cold seasons. In the hot season, the RRs of CRD hospitalization increased rapidly in the relatively low DTR (Fig. 2). Heatwaves are appeared in the hot season with extreme low DTR. The adverse effects of Heatwaves on health have been widely confirmed. We conjecture that the adverse effects of low DTR in hot season may be related to the heatwaves [37]. However, insignificant effects of extremely low DTR were found in the cold season. Given the minimum temperature in the study period is 2.0 °C (Table 1), the adverse effect of cold spells was insignificant on hospitalization for CRD in Guangdong Province. Higher RRs in moderate and high DTR were observed in the cold season, demonstrating the importance of developing the preventive measures of adapting to large DTR in the cold season. For example, it is needed to provide home heating and timely clothing for large DTR in the cold season [38].

The associations of DTR with adverse outcomes of COPD patients have been confirmed in previous studies. A time-series analysis conducted in Shanghai city reported that the association between DTR and daily COPD mortality was significant [39]. The emergency room visit for exacerbations of COPD was associated with DTR in an ecological study in Taichung city, Taiwan [31]. However, a city-level time series analysis reported that the insignificant relationship between DTR and hospitalization for total COPD patients was found in Changchun, a northeastern city of China [21]. Using data from 21 cities and the method of meta-analysis, we demonstrated that the RR of hospital admissions for exacerbations of COPD was 1.11 (95%CI: 1. 08 to 1.12) at the 75th percentile compared to the 25th percentile of DTR at lag0–6. Furthermore, a previous study in Changchun city observed the greatest estimates for males appeared at lag 7 days, which is in line with our maximum estimate at lag0–6. Several single-city studies have observed the association of DTR with adverse health outcomes, including emergency room visits and hospital admissions, of asthma patients [9, 40, 41]. Our study discovered that the maximum lag effect of DTR on asthma exacerbations was at lag0–6 and the changed slightly until lag0–14. Similar effects were found on emergency department admissions in Brisbane, Australia [40]. To our knowledge, the relationship between hospitalization for bronchiectasis and DTR was assessed firstly. No significant association between DTR and total bronchiectasis patients was found. However, we found that DTR was a risk factor for female bronchiectasis patients who has a higher rate of hospitalization than male patients [42]. Both exacerbations of COPD and asthma have been confirmed to be associated with DTR, but their associations have not been compared directly. The subgroup analysis on diverse CRD shows that COPD patients are most vulnerable to D TR (RR = 1.11[95%CI: 1.08 to 1.1]), and bronchiectasis patients are not sensitive to the temperature change within a day. These results enhanced the importance of reducing the adverse impacts of DTR on CRD patients, especially COPD patients.

To the best of our knowledge, this is the first multi-city study to examine the short-term effect of the DTR on daily hospitalization of CRD. However, there are several limitations of our study. Firstly, measures of DTR were mainly obtained from 21 fixed-site monitoring stations (the data of Chaozhou city and Foshan city was replaced by nearest monitoring site) rather individual exposure. Using city-wide meteorological measures could lead to exposure measurement errors that underestimate the adverse effect of the temperature variation within a day [43]. Secondly, the hospitalization data of each patient depend on the hospital address, not exactly the individual living region. We assumed people would go to the hospital near their living region in a critical situation, and the exposure measurement errors for inter-cities patients could not be solved. Thirdly, although we obtained data from fixed 227 hospitals, the existing CRD populations might have increased due to the aging process of society, which could influence our estimates.

Our findings may close the knowledge gap of the relationship between DTR and CRD and highlight the importance of preventive measures, such as providing home heating, suitable clothing for large DTR, staying indoor to avoid environment temperature variation.

## Conclusion

Our study observed the independent effect of DTR on hospitalization for CRD (i.e., COPD, asthma and bronchiectasis). The effects of DTR on hospital admissions for CRD were strong at low DTR of the hot season and high DTR of the cold season. COPD and asthma patients were more vulnerable to DTR than bronchiectasis patients. The adverse effect of DTR on hospital admissions for bronchiectasis was only observed in female patients. Preventive measures to reduce the adverse impacts of DTR were needed for CRD patients.

## Supplementary information

**Additional file 1.**

## Data Availability

All data which were generated or analysed are included in this published article [and also its supplementary information files].

## Abbreviations

COPD: Chronic obstructive pulmonary disease
DTR: Diurnal temperature range
CRD: Chronic respiratory diseases
