# Supplementary material for: Association of diurnal temperature range with daily hospitalization for exacerbation of chronic respiratory diseases in 21 cities, China
Source: Respir Res. 2020 Sep 29;21:251. doi: 10.1186/s12931-020-01517-7 (PMC7526384; doi:10.1186/s12931-020-01517-7)
Supplement: Supplementary file 1 — Additional file 1. [file 12931_2020_1517_MOESM1_ESM.pdf]

e-Figure 1. 21 cities in Guangdong Province.

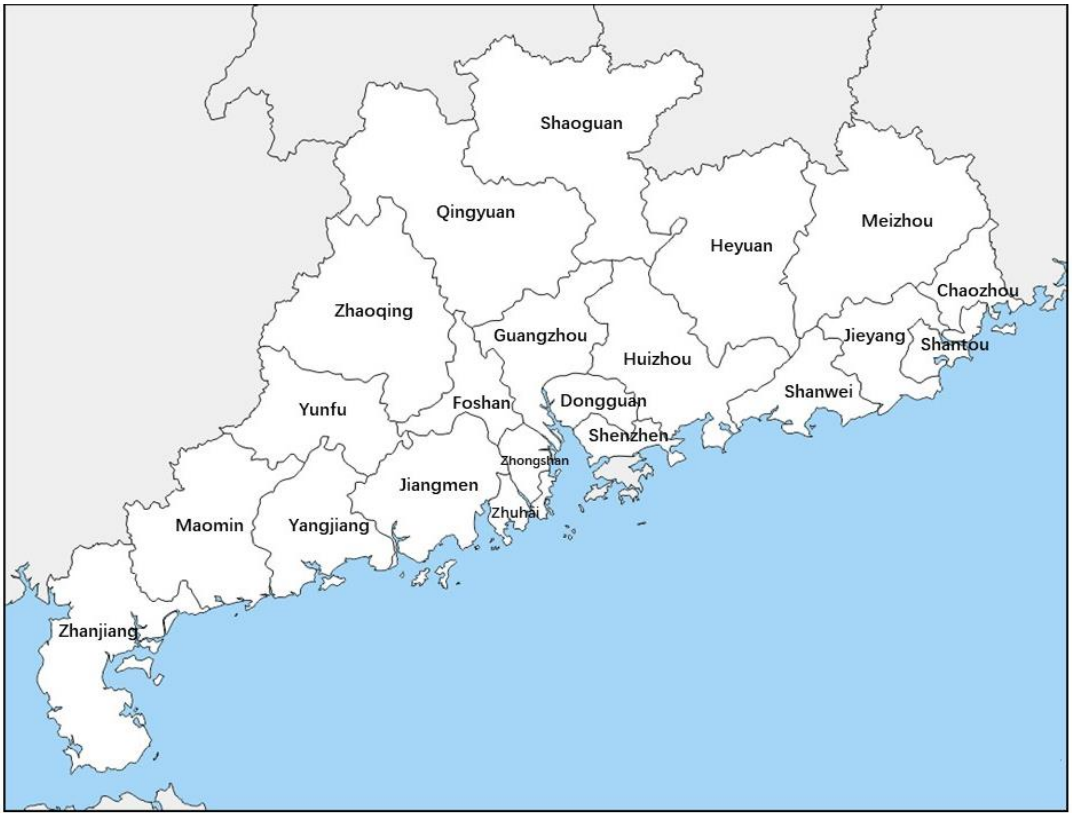

e-Table 1. DTR level for 21 cities

	Min	25th	50th	75th	Max
Chaozhou	1.6	5.7	8.0	10.3	20.1
Dongguan	1.0	5.0	7.0	8.0	14.0
Foshan	1.0	5.6	7.8	9.8	18.6
Guangzhou	1.0	5.5	8.0	10.0	18.5
Heyuan	1.0	6.0	8.7	10.8	20.7
Huizhou	1.0	6.0	8.0	9.0	15.0
Jiangmen	1.0	4.0	5.5	6.5	12.5
Jieyang	1.5	5.5	7.0	9.0	17.0
Maoming	1.0	5.5	7.0	9.0	18.5
Meizhou	1.0	6.4	9.0	11.3	21.3
Qingyuan	1.2	5.4	7.7	9.8	19.7
Shantou	0.0	4.5	6.0	7.0	16.0
Shanwei	0.0	4.0	5.0	7.0	15.0
Shaoguan	1.0	5.3	8.3	11.0	21.4
Shenzhen	1.0	5.0	6.0	7.0	14.0
Yangjiang	1.0	4.0	6.0	7.0	14.0
Yunfu	1.0	6.0	8.0	10.0	19.0
Zhanjiang	0.5	5.0	6.5	8.0	14.0
Zhaoqing	1.0	5.5	8.0	9.5	18.5
Zhongshan	1.0	5.0	7.0	8.0	17.0
Zhuhai	1.0	4.0	5.0	6.0	12.0

e-Table 2. City-specific relative risks (95% CI) and heterogeneity from random-effect meta-analysis*

	COPD	Asthma	Bronchiectasis	Chronic respiratory disease
Chaozhou	0.97 (0.82 to 1.16)	1.09 (0.75 to 1.59)	1.05 (0.71 to 1.53)	1.00 (0.86 to 1.16)
Dongguan	1.11 (1.03 to 1.20)	1.21 (1.04 to 1.40)	0.98 (0.88 to 1.10)	1.09 (1.03 to 1.16)
Foshan	1.09 (1.04 to 1.15)	1.01 (0.91 to 1.11)	1.00 (0.93 to 1.07)	1.06 (1.02 to 1.11)
Guangzhou	1.08 (1.04 to 1.13)	1.09 (1.00 to 1.19)	1.04 (0.97 to 1.10)	1.07 (1.03 to 1.12)
Heyuan	1.07 (0.98 to 1.16)	1.21 (0.93 to 1.58)	1.04 (0.80 to 1.35)	1.08 (0.99 to 1.17)
Huizhou	1.11 (1.05 to 1.18)	0.98 (0.83 to 1.16)	1.20 (1.03 to 1.39)	1.11 (1.05 to 1.18)
Jiangmen	1.20 (0.99 to 1.45)	0.88 (0.54 to 1.42)	0.76 (0.50 to 1.15)	1.08 (0.91 to 1.27)
Jieyang	1.14 (1.03 to 1.26)	1.22 (0.99 to 1.52)	0.84 (0.67 to 1.06)	1.10 (1.01 to 1.21)
Maoming	1.09 (1.03 to 1.15)	1.14 (1.01 to 1.28)	1.01 (0.90 to 1.12)	1.09 (1.04 to 1.14)
Meizhou	1.03 (0.95 to 1.11)	1.16 (0.93 to 1.44)	1.21 (0.98 to 1.50)	1.06 (0.99 to 1.13)
Qingyuan	1.15 (1.09 to 1.21)	1.04 (0.83 to 1.31)	0.92 (0.79 to 1.07)	1.13 (1.07 to 1.18)
Shantou	1.10 (0.99 to 1.21)	1.14 (0.96 to 1.35)	1.01 (0.83 to 1.21)	1.09 (1.01 to 1.19)
Shanwei	1.16 (1.02 to 1.33)	1.08 (0.73 to 1.59)	0.90 (0.63 to 1.27)	1.12 (0.99 to 1.27)
Shaoguan	1.14 (1.07 to 1.20)	1.11 (0.96 to 1.27)	1.21 (1.05 to 1.39)	1.14 (1.08 to 1.20)
Shenzhen	1.04 (0.96 to 1.12)	1.09 (0.97 to 1.23)	1.04 (0.93 to 1.15)	1.05 (0.99 to 1.11)
Yangjiang	1.12 (1.00 to 1.26)	1.02 (0.81 to 1.29)	0.85 (0.66 to 1.10)	1.07 (0.97 to 1.18)
Yunfu	1.07 (0.98 to 1.16)	0.98 (0.71 to 1.35)	0.97 (0.72 to 1.31)	1.06 (0.98 to 1.14)
Zhanjiang	1.06 (1.00 to 1.12)	1.22 (1.04 to 1.43)	0.92 (0.81 to 1.05)	1.05 (1.00 to 1.10)
Zhaoqing	1.10 (1.03 to 1.17)	0.95 (0.79 to 1.14)	1.07 (0.91 to 1.25)	1.08 (1.02 to 1.15)
Zhongshan	1.11 (1.05 to 1.17)	1.04 (0.89 to 1.23)	1.02 (0.92 to 1.14)	1.09 (1.04 to 1.14)
Zhuhai	1.03 (0.61 to 1.74)	0.55 (0.16 to 1.85)	1.17 (0.59 to 2.30)	1.00 (0.67 to 1.49)
I^2 (%)	36.61%	6.90%	1.01%	31.92%
Q test (p-value)	0.001	0.304	0.480	0.004

* Results are presented by relative risk (95% CI) at 75th percentile compared to 25th percentile of DTR at lag0-6.

e-Table 3. Sensitivity analysis: relative risks (95% CI) of hospitalizations for exacerbations of chronic respiratory disease.*

Variables	relative risks (95% CI)
Numbers of moving average for diurnal temperature range	
1 (lag 0)	1.02 (1.01 to 1.02)
3 (lag 0-2)	1.04 (1.03 to 1.06)
5 (lag 0-4)	1.07 (1.06 to 1.09)
7 (lag 0-6)	1.09 (1.07 to 1.10)
14 (lag 0-13)	1.09 (1.07 to 1.11)
21 (lag 0-20)	1.09 (1.06 to 1.12)
Degree of freedom for time per years	
6	1.07 (1.06 to 1.09)
7	1.07 (1.05 to 1.08)
8	1.09 (1.07 to 1.10)
9	1.08 (1.07 to 1.10)
10	1.09 (1.08 to 1.10)
Degree of freedom for meteorological measures	
3	1.09 (1.07 to 1.10)
6	1.09 (1.07 to 1.10)
Spline function	
natural cubic spline function	1.09 (1.07 to 1.10)
penalized splines function	1.08 (1.07 to 1.10)
Dataset	
cities with > 5 hosiptals data	1.08 (1.07 to 1.10)
21 cities	1.09 (1.07 to 1.10)

* Results are presented by relative risk (95% CI) at 75th percentile compared to 25th percentile of DTR at lag0-6.
